# Molecular analysis of local relapse in high-risk breast cancer patients: can radiotherapy fractionation and time factors make a difference?

**DOI:** 10.1038/sj.bjc.6600755

**Published:** 2003-03-04

**Authors:** M I Koukourakis, A Giatromanolaki, G Galazios, E Sivridis

**Affiliations:** 1 Departments of Radiotherapy/Oncology, Democritus University of Thrace, Alexandroupolis 68100, Greece; 2 Departments of Pathology, Democritus University of Thrace, Alexandroupolis 68100, Greece; 3 Department of Gynecology, Democritus University of Thrace, Alexandroupolis 68100, Greece

**Keywords:** breast cancer, radiotherapy fractionation, angiogenesis, c-erbB-2

## Abstract

Large primary breast tumours and extensive lymph node involvement are linked to a high rate of local recurrence after surgery. In 10–20% of such high-risk breast cancer patients, local relapse will occur despite postoperative radiotherapy. In the present study, we investigated whether molecular features, such as angiogenesis, cancer cell proliferation, steroid receptor expression, c-erbB-2 oncoprotein overexpression, p53 protein nuclear accumulation or bcl-2 antiapoptotic protein expression, can predict failure of local therapy. We further examined as to which subgroups of patients could benefit from altered fractionation schemes of radiotherapy. In univariate analysis, high intratumoural angiogenesis, c-erbB overxpression and mutant-p53 nuclear accumulation were significantly associated with increased relapse rate (*P*=0.0002, 0.009 and 0.05, respectively). In multivariate analysis, the microvessel density and the c-erbB-2 status were independent and significant factors related to local relapse (*P*=0.001, *t*-ratio 3.36 and *P*=0.02, *t*-ratio 2.26, respectively). Hypofractionated and accelerated radiotherapy supported with amifostine (HypoARC regimen) was significantly more effective than standard radiotherapy in cases with high cancer cell proliferation index, c-erbB-2 and p53 overexpression. High angiogenesis, however, was linked with local relapse regardless of the radiotherapy regimen.

Adjuvant radiotherapy significantly improves the local control rates even after radical or partial mastectomy in patients with aggressive tumour features, such as large primary tumours or extensive node involvement (Forrest *et al*, 1996; Early Breast Cancer Trialists Collaborative Group, 1995; Griem *et al*, 1987; Velez-Garcia *et al*, 1992). Although controversial, several large studies suggested that local control is also important for the overall survival of patients (Overgaard *et al*, 1997,^1999^; Ragaz *et al*, 1997; Clark *et al*, 1996). As local recurrence after surgery (with or without chemotherapy) is less than 20% (Mcneil, 1999; Recht *et al*, 1999), radiotherapy is often omitted (Athas *et al*, 2000) as it is difficult to weigh the benefits of reduced cost and patient inconvenience that result from the elimination of postoperative radiotherapy against the disadvantages of local recurrence (Liljegren *et al*, 1994).

A recent study reported the results of a prospective study on 72 breast cancer patients with features of locally aggressive or advanced disease treated with surgery, FEC (5-fluorouracil 500 mg m^−2^, epirubicine 60 mg m^−2^ and cyclophosphamide 500 mg m^−2^) chemotherapy and a very accelerated scheme of hypofractionated radiotherapy supported with high-dose daily amifostine (Koukourakis *et al*, 2002a). The whole radiotherapy dose is given within 12 consecutive radiotherapy fractions (16 days). After a median of 28 months of follow-up (range 18–42 months), we concluded that the regimen is well tolerated with significantly reduced early radiation sequelae and with late toxicity (breast, axilla, thoracic wall) at least equal to that observed in control groups of patients, within the same follow-up time.

Such a regimen is certainly convenient for patients and radiotherapy departments, as it reduces the overall days of radiotherapy down to 35% of the days required for a 45-day conventional fractionation regimen. Nevertheless, the rationale for applying such an aggressive regimen was also based on a potential advantage in terms of local control. Hypofractionated radiotherapy regimen supported with amifostine cytoprotection (HypoARC), theoretically, could be more effective for tumours for several reasons described in detail in previous papers (Koukourakis *et al*, 2002a; Koukourakis and Yannakakis, 2001). Briefly, radiotherapy acceleration could abrogate the ominous effect of ‘rapid tumour repopulation’ on the efficacy of radiotherapy. Furthermore, the use of large radiotherapy fractions could prove more effective in tumours with low intrinsic radiosensitivity. As hypoxia pushes the *β*-value of the *α*/*β* ratio to higher levels (Chapman *et al*, 1975), hypoxic tumours may be resistant to low dose per fraction used in standard or hyperfractionated radiotherapy. In that way, HypoARC could be more effective in hypoxic breast tumours. On the other hand, the addition of high-dose amifostine before each radiotherapy fraction could help in overcoming the severe early and late toxicity expected from such an aggressive radiotherapy regimen, which is indeed confirmed in a prospective study (Koukourakis *et al*, 2002a). Local relapse analysis, however, did not show a significant benefit over standard radiotherapy, although the 1- and 2-year local relapse-free survival (LRFS) was 95 and 84% in the HypoARC group, respectively, *vs* 84 and 76% in the standard radiotherapy arm (Koukourakis *et al*, 2002a).

In the present study, we show that specific molecular features relevant to rapid tumour repopulation or to intrinsic radiosensitivity (i.e. high proliferation index, p53-mutations or c-erbB-2 overexpression) are linked with high local relapse rate following postoperative radiotherapy, which can be averted by using large radiotherapy fractions and treatment acceleration. High angiogenesis, however, predicts a high rate of local relapse independent of the radiotherapy scheme.

## MATERIALS AND METHODS

From March 1997 to April 1999 at the University Hospital of Heraklion, 72 high-risk breast cancer patients treated with surgery and adjuvant FEC chemotherapy were recruited in a large phase II study in order to assess the toxicity and efficacy of HypoARC as an adjuvant regimen (Koukourakis *et al*, 2002a). Inclusion and exclusion criteria have been previously reported. Briefly, high-risk breast cancer patients were recruited (T3,4-stage and/or metastatic disease in more than three lymph nodes), and all patients had a good performance status with no other major disease. Informed consent was obtained before inclusion.

The survival of patients treated with adjuvant HypoARC chemotherapy was compared to that of a matched control group of 59 breast cancer patients treated with modified radical mastectomy followed by six cycles of FEC chemotherapy and conventionally fractionated radiotherapy. These patients were treated at the same department, during the same period and fulfilled the criteria used for the recruitment of patients in the HypoARC study. Patients' characteristics and tumour stage were similar in both groups (Table 1

**Table 1:** Patients' and tumour characteristics

**Parameter**	**HypoARC (31 patients)**	**CRT (40 patients)**
*Surgery*		
Mastectomy	10	17
Conservative	21	23
*Surgical margins*		
Negative	31	40
Positive	0	0
*Tumor focality*		
Unifocal	31	40
Multifocal	0	0
*T-N stage*		
T1-N1b _iii,iv_,2	3	6
T2-N1b _i,ii_	8	12
T2- N1b _iii,iv_,2	15	15
T3-N1b,2	5	7
*Grade*		
1,2	11	19
3	2	21
*Menopausal status*		
Pre	10	15
Post	21	25

). All tumours were of the ductal type, unifocal and the surgical margins were negative as revealed in the histopathology report.

Archieval paraffin-embedded material from the primary breast carcinoma was retrieved from hospitals, where the surgery had been performed. We managed to collect tissue samples from 31 out of 72 patients treated with HypoARC and from 40 out of 59 patients treated with conventional radiotherapy (CRT). Failure to collect additional samples was mainly because of pathological diagnosis in centres outside the island of Crete and because of exhausted tissue material (used in other research projects).

### Radiotherapy details

The same irradiation technique was used for standard radiotherapy and HypoARC. Field positioning was performed with the aid of a simulator following computerised planning. The breast (or chest wall), axilla and supraclavicular area were treated with a LINAC 6MV. Tangential fields were used to irradiate the breast or chest wall and special care was taken during the simulation so that less than 2 cm of thickness of normal lung was included in these fields. The inner borders were placed 2 cm away from the sternal midline while the outer border followed the middle axillary line. Supraclavicular and axillary areas were irradiated with an anterior field, the dose being calculated at 4 cm of depth. A booster posterior field directed to the axilla (dose calculation at 7 cm) was treated immediately following the treatment of the anterior field in order to compensate for the loss of dose because of increased depth. The dose administered with this posterior field was 15% of the dose given with the anterior one. Tangential fields were also used to deliver a booster dose to the tumour bed, where a 1 cm tissue equivalent material was placed over this area (5–8 cm × 7–10 cm) in order to increase the dose to the surface. The onset of radiotherapy was 25–30 days after the delivery of the last cycle of chemotherapy. Table 2

**Table 2:** Radiotherapy schedules used in HypoARC and in the control group

	**Field**	**(Gy/fraction) × (No fractions)**	**Physical dose (Gy)**	**Overall treatment time (days)**
*HypoARC*				16
Chest wall/breast	Tang	3.5 × 4	14	
	Tang	4 × 6	24	
	*Total*		38	
Supraclavicular/axillary	a+p	3.5 × 11	38.5	
Tumour bed (boost)	Tang	4 × 2	8	
	*Total*		46	
*Standard RT*				44
Chest wall	Tang	2 × 25	50	
	*Total*		50	
Tumour bed	Tang	2 × 7	14	
	*Total*		64	
Supraclavicular/axillary	a+p	2 × 25	50	

Tang=tangential fields; a+p=0 anterior and posterior fields.

shows the radiotherapy fractionation used. Radiobiological analysis has been reported in a previous study (Koukourakis *et al*, 2002a).

### Administration of amifostine

Tropisetron (10 mg i.v., in 50 ml NS infused within 5 min) was given as antiemetic premedication. Amifostine (Ethyol®), 1000 mg diluted in 50 ml NS, was rapidly infused within 5 min, the patient being in a supine position, and under continuous monitoring of blood pressure. No steroids were used unless indicated by severe vomiting. Radiotherapy was delivered 20 min after the amifostine infusion.

### Immunohistochemical studies

A modified streptavidin immunohistochemical technique was used to assess the expression of different proteins and oncoproteins. Sections were dewaxed and peroxidase was quenched with methanol and H_2_O_2_ 3% for 15 min. Microwaving for antigen retrieval was used (3 × 4 min). The primary antibodies were applied for 90 min. Following washing with TBS, sections were incubated with a secondary rabbit-anti-mouse antibody (Kwik Biotinylated Secondary, 0.69A Shandon-Upshaw) for 15 min and washed in TBS. Kwik Streptavidin peroxidase reagent (039A Shandon-Upshaw) was applied for 15 min and sections were again washed in TBS. The colour was developed by 15 min incubation with DAB solution and sections were weakly counterstained with haematoxylin. Normal mouse immunoglobulin-G was substituted for primary antibody as the negative control (same concentration as the test antibody), while appropriate tissue samples were used for positive controls.

For the assessment of the proliferation index, the MIB-1 MoAb (clone PRO224, YLEM, Italy) was used. The percentage of cells with nuclear reactivity was recorded in all fields and the mean value was calculated. The median MIB-1 score was used as a cutoff point to define two groups of tumours with low *vs* high proliferation index.

For c-erbB-2 expression, the NCL-CB11 MoAb was used (Novocastra Laboratories, UK). The percentage of cancer cells with a clear membrane staining was recorded in all optical fields and the mean value was calculated. Tissue samples with a mean value >20% were considered as positive for c-erbB-2 protein overexpression.

For mutant p53 protein expression, we used the DO7 MoAb (DAKO, Denmark). The percentage of cancer cells with nuclear reactivity was recorded in all optical fields and the mean value was calculated. Tumour samples with a percentage of positive cancer cells >10% were considered as positive for p53. Using a similar method of assessment, the nuclear expression of oestrogen and progesterone receptors was examined by applying the mouse MoAb clone 1D5 and the clone 1A6 (dilution 1 : 100; Immunon-Shandon, USA), respectively. A cutoff point of 10% of reactive cells was also used for positivity.

For bcl-2 protein expression, the clone 100 MoAb (DAKO, Denmark) was used. The percentage of cancer cells with strong cytoplasmic and/or perinuclear expression was recorded in all optical fields and the mean value was calculated. Tumour samples with a mean value higher than 10% were considered as positive for bcl-2 expression.

### Assessment of the intratumoural microvessel density (MVD)

The JC70 monoclonal antibody (DAKO, Denmark) recognising the CD31 (platelet/endothelial cell adhesion molecule; PECAM-1) was used for microvessel staining on 2 *μ*m paraffin-embedded sections using the alkaline phosphatase/antialkaline phosphatase (APAAP) procedure. Sections were dewaxed, rehydrated and predigested with protease type XXIV for 20 min at 37°C. The JC70 (1 : 20) was applied at room temperature for 30 min and washed in TBS. Rabbit-anti-mouse antibody 1 in 50 was applied for 30 min, followed by application of APAAP complex 1 in 1 for 30 min. After washing in TBS, the last two steps were repeated for 10 min each. The colour was developed by 20 min incubation with New Fuchsin solution. Sections from primary tumours were scanned at low power (×40 and ×100).

The areas of the highest vascularisation were chosen at low power (×100) and microvessel counting followed on three chosen ×200 fields of the highest density (hot spots). Microvessels adjacent to normal breast or around necrotic areas were excluded from the appraisal. The MVD was the mean of the vessel counts obtained. The median MVD was used to define two groups of patients with low *vs* high MVD. Vessels with a clearly defined lumen or well-defined linear vessel shape but not single endothelial cells were taken into account for microvessel counting.

### Statistical analysis

Statistical analysis and graphs were performed using the GraphPad Prism 2.01 package. A Fisher's exact test or the unpaired two-tailed *t*-test was used for testing relations between the categorical variables, as appropriate. Local relapse and overall curves were plotted using the method of Kaplan–Meier, and the log-rank test was used to determine statistical differences between life tables. A Cox proportional hazard model was used to assess the effects of patient and tumour variables on overall survival. A *P*-value of <0.05 was considered significant.

## RESULTS

### Results of immunohistochemistry

The results of immunohistochemistry in the two groups of patients (HypoARC *vs* CRT) are shown in Table 3

**Table 3:** Results of molecular analysis according to the type of radiotherapy

**Parameter**	**HypoARC (31 patients)**	**CRT (40 patients)**
*MVD*		
Low	16	20
High	15	20
		
*MIB-1 index*		
Low	19	28
High	12	12
		
*c-erbB-2*		
Negative	21	33
Positive	10	7
		
*mt-p53*		
Negative	21	29
Positive	10	11
		
*bcl-2*		
Negative	8	18
Positive	23	22
		
*ER*		
Negative	12	17
Positive	19	23
		
*PgR*		
Negative	21	29
Positive	10	11

. Overall, 35 out of 71 patients had high intratumoural MVD, 24 out of 71 high MIB-1 proliferation index, 17 out of 71 had membrane c-erbB-2 protein overexpression, 21 out of 71 had nuclear mt-p53 protein accumulation and 45 out of 71 had positive cytoplasmic bcl-2 expression. Oestrogen and progesterone receptor expression in the cancer cell nuclei was noted in 42 out of 71 and 21 out of 71 tumours, respectively. The immunohistochemical findings were well balanced between the two groups, but the bcl-2 expression was more frequently noted in the group of patients treated with HypoARC.

### Local relapse-free survival (LRFS) analysis

**Figure 1:**
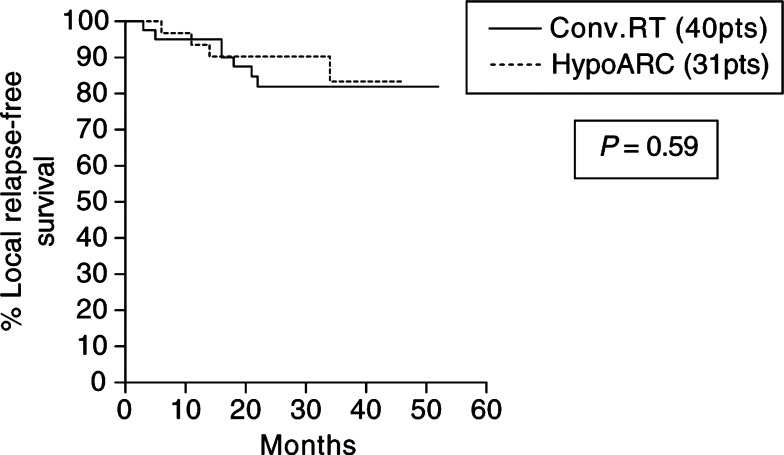
Kaplan–Meier survival curves stratified for radiotherapy schedule (conventional *vs* HypoARC).

Out of 31 patients treated with HypoARC, four (12.9%) relapsed locally *vs* seven out of 40 (17.6%) of patients treated with CRT. The LRFS of patients treated with HypoARC or CRT is plotted in Figure 1

. There was no significant difference between the two groups (*P*=0.59). The type of surgery (mastectomy *vs* tumourectomy) or the menopausal status was not related to local relapse (*P*=0.40 and 0.96, respectively; data not shown).

Table 4

**Table 4:** Univariate and multivariate analyses of local relapse: histological and molecular variables

		**Univariate**			**Multivariate**		
		***P*-value**	**Risk ratio**	**95%CI**	***P*-value**	**Risk ratio**	**95% CI**
T-stage	T3 *vs* T1,2	0.13	1.95	0.3–8.4	0.22	1.23	0.4–2.1
N-stage	N1biii,iv,2 *vs* N0,1bi,ii	0.84	1.10	0.3–3.8	0.51	1.41	0.6–2.6
Grade	3 *vs* 1,2	0.20	1.90	0.5–6.1	0.42	1.25	0.5–2.5
MVD	High *vs* low	0.0002	16.0	11–24	0.001	3.36	2.1–9.8
MIB-1	High *vs* low	0.11	2.42	0.7–9.2	0.46	1.38	0.5–2.1
c-erbB-2	Positive *vs* negative	0.009	4.20	1.6–18.7	0.02	2.26	1.5–6.9
mt-p53	Positive *vs* negative	0.05	3.15	1.0–14	0.12	1.56	0.9–4.8
bcl-2	Negative *vs* positive	0.18	1.95	0.6–8.1	0.32	0.98	0.4–2.9
ER	Negative *vs* positive	0.32	1.80	0.5–6.2	0.74	0.32	0.1–1.1
PgR	Negative *vs* negative	0.37	1.80	0.4–6.2	0.99	0.01	0–0.1
RT-regimen	Hypoarc *vs* Standard	0.55	0.71	0.2–2.3	0.38	0.36	0.1–1.4

shows the univariate and multivariate analyses of LRFS in all patients considered together. In univariate analysis, high intratumoural angiogenesis, c-erbB overxpression and mutant-p53 nuclear accumulation were significantly associated with increased relapse rate (*P*=0.0002, 0.009 and 0.05, respectively). In multivariate analysis, the MVD and the c-erbB-2 status were independent and significant factors related to local relapse (*P*=0.001, *t*-ratio 3.36 and, *P*=0.02, *t*-ratio 2.26, respectively).

### Local relapse-free survival LRFS analysis according to the radiotherapy regimen

The Kaplan–Meier LRFS curves were plotted and the log-rank test was calculated, using double stratification for the radiotherapy method and for each one of the assessed histological and molecular variables.

**Figure 2:**
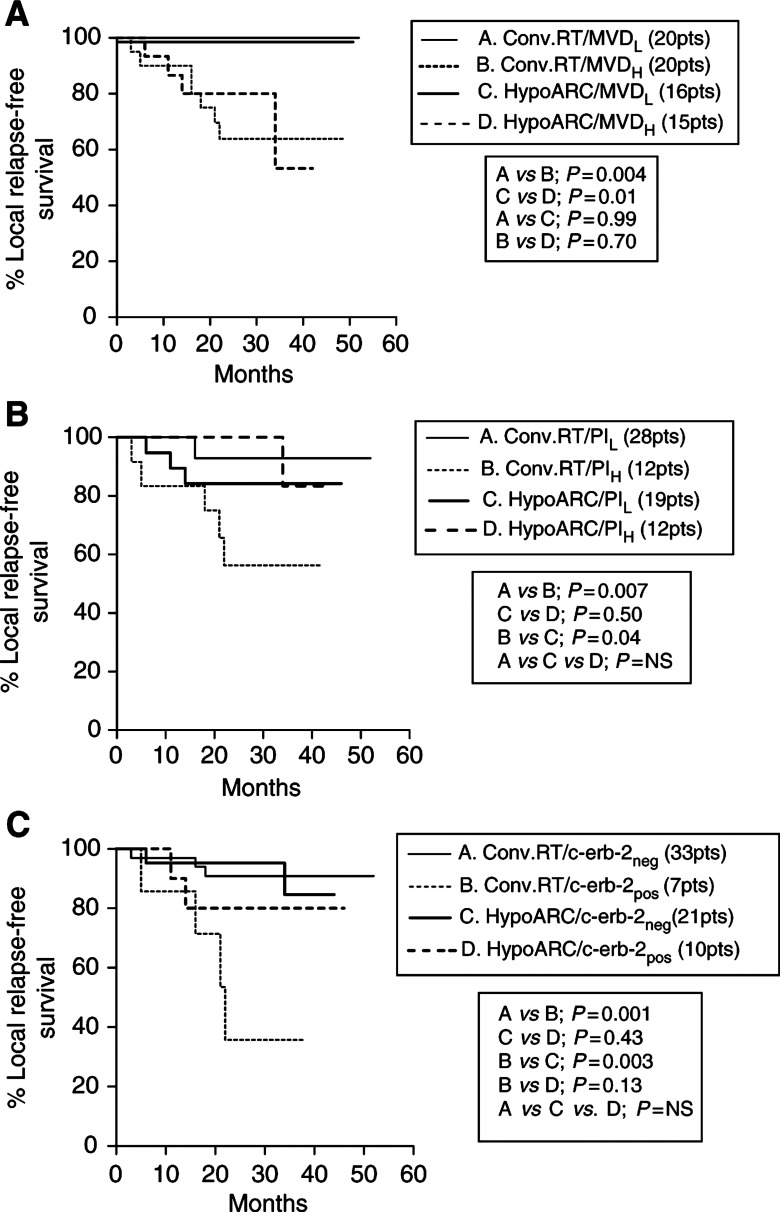
(**A**) Kaplan–Meier survival curves stratified for radiotherapy schedule (conventional *vs* HypoARC) and microvessel density (low *vs* high). (**B**) Kaplan-Meier survival curves stratified for radiotherapy schedule (conventional *vs* HypoARC) and MIB1 proliferation index (low *vs* high). (**C**) Kaplan–Meier survival curves stratified for radiotherapy schedule (conventional *vs* HypoARC) and c-erbB-2 membrane overexpression (negative *vs* positive).

High MVD was associated with poor LRFS whether patients were treated with HypoARC or CRT (Figure 2A

). On the contrary, high proliferation MIB-1 index was associated with significantly poor LRFS in patients treated with CRT (*P*=0.007), but not in patients treated with HypoARC (*P*=0.50). Patients with high proliferation index treated with HypoARC had a significantly better LRFS than patients treated with CRT (*P*=0.04); Figure 2B.

Similarly, patients overexpressing c-erbB-2 had a significantly poorer LRFS when treated with CRT (*P*=0.001), while c-erbB-2 status was not of predictive significance in patients treated with HypoARC. The LRFS of c-erbB-2+ patients treated with HypoARC was better, although not significantly, than the one of c-erbB-2+ patients treated with CRT (*P*=0.13); Figure 2C.

Nuclear expression of mt-p53 was associated with poor LRFS only in the group of patients treated with CRT (*P*=0.002). Patients with mt-p53+ tumours treated with HypoARC had a better LRFS than patients with mt-p53+ tumours treated with CRT (*P*=0.06).

Analysis of T-stage, N-stage and histology grade did not reveal any significant differences between the HypoARC and the CRT efficacy in preventing local relapse. Similarly, HypoARC and CRT were equally effective within the positive or negative for ER, PgR or bcl-2 subgroups of patients (data not shown).

Multivariate analysis within the group of patients treated with CRT revealed that high MVD (*P*=0.009, *t*-ratio 2.76), c-erbB-2 overexpression (*P*=0.05, *t*-ratio 1.98) and mt-p53 expression (*P*=0.05, *t*-ratio 1.96) were independent variables related to local relapse. Multivariate analysis within the HypoARC group of patients showed that only high MVD had an independent predictive role of local relapse, although of marginal significance (*P*=0.07, *t*-ratio 1.86).

## DISCUSSION

An important role of molecular variables in defing relapse of breast cancer after local and systemic therapy has been raised during the past decade. Loss of oestrogen or progesterone receptor expression was the first molecular feature recognised to associate with poor survival in breast cancer (Chevallier *et al*, 1988). C-erbB-2 expression has been linked to resistance to chemotherapy and poor outcome (Andrulis *et al*, 1998), while apoptosis-related proteins such as p53 and bcl-2 have been related to disease-free interval and survival of breast cancer patients (Thor *et al*, 1992; Gasparini *et al*, 1995). Similarly, a large body of clinicopathological studies support the important and independent prognostic role of the tumour angiogenic ability in operable breast cancer (Locopo *et al*, 1998). Most of the studies, however, focus on the overall relapse events, while the impact of molecular variables on the efficacy of postoperative radiotherapy to control local disease remains largely unknown.

Nevertheless, several histopathological or patient-related variables have been reported to define a higher risk of local relapse after postoperative radiotherapy. In a recent analysis by Katz *et al* (2001), in 1031 mastectomised breast cancer patients, disease close to the surgical margins, gross multifocality, tumour size >5 cm and cancer metastasis in more than three axillary nodes were independently related to a higher risk of local relapse following postoperative radiotherapy. Similar results have been reported in another recent study from Japan, in 301 stage I/II breast-cancer patients treated with lumpectomy or quadrantectomy (Kodaira *et al*, 2001). Young age and less extensive surgery had an independent predictive role of local relapse, while positive surgical margins or extensive intraductal component had a marginal significance. Systemic therapy seems also to contribute to the local relapse-free interval of breast-cancer patients, as recently shown in a study of 484 node negative breast cancer patients treated with breast conservative surgery and radiotherapy (Buchholz *et al*, 2001).

Our study comprised a relatively limited number of breast cancer patients. All of them, however, although operable had local and/or regional extensive disease. This was a result of the inclusion criteria used for the enrolment of patients in the HypoARC study (Koukourakis *et al*, 2002a). All patients had received FEC chemotherapy before radiotherapy. Clear surgical margins and unifocal disease was a prerequisite to include a tumour sample in the present clinicopathological study. Thus, out of the traditional patient/histology variables, menopausal status and surgical technique were the only ones left to test as predictors of local relapse. None of them was significantly related to local relapse in this high-risk series of patients.

Molecular analysis, however, showed that increased intratumoural MVD, c-erbB-2 membrane overexpression, accumulation of mutant p53 protein and high proliferation index assessed with the MIB-1 MoAb defines subgroups of patients with increased risk of local relapse, in high-risk breast cancer patients. In multivariate analysis, high MVD and c-erbB-2 overexpression retained their significant and independent predictive role for local relapse, while p53 and proliferation index did not.

The important role of upregulated angiogenic pathways in the outcome of radiotherapy has been recently raised (Koukourakis *et al*, 2000a,2000b). It seems that high angiogenic activity confers a proliferation/apoptosis advantage in cancer cells during radiotherapy (Matsushita *et al*, 1999; Natsura *et al*, 1999), while an angiogenic regeneration of tumours during radiotherapy cannot be excluded (Koukourakis *et al*, 2000b). C-erbB-2 protein overexpression in breast cancer seems also to relate to resistance to radiotherapy (Formenti *et al*, 2002). Treatment of cancer cells with anti-c-erbB-2 MoAbs before radiotherapy significantly enhances the cytotoxic effect of radiotherapy, which shows that c-erbB-2 overexpression is involved in biological pathways relevant to the intrinsic radiosensitivity (Pietras *et al*, 1999). c-erbB-2 involvement in the upregulation of the VEGF angiogenic pathway (Petit *et al*, 1997) suggests that c-erbB-2-related radioresistance is, at least in part, linked to the tumour angiogenic activity. The involvement of wild-type p53 protein in the radiation-induced apoptosis (Brugarolas *et al*, 1995), at least in part, explains the finding that mutant p53 expression is related to reduced efficacy of local radiotherapy. A strong association of nuclear p53 protein accumulation with local relapse in breast cancer patients receiving or not postoperative radiotherapy has been recently reported by Zellars *et al* (2000). The association of a high proliferation index with local relapse may be explained by a possible role of rapidly proliferating cells in the so-called ‘accelerated clonogen repopulation’ phenomenon during radiotherapy (Withers *et al*, 1998). An increased rate of locoregional and distant relapse has been reported in patients with breast cancer bearing a high thymidine labelling index (Amadori and Silvestrini, 1998).

About half of the cases included in the study were treated with an accelerated HypoARC. Analysis of local relapse within the groups of patients treated with CRT and HypoARC radiotherapy showed a striking difference regarding the influence of the examined molecular variables. High proliferation index was not a predictive factor of local relapse in the group of patients treated with accelerated (HypoARC) radiotherapy. The 2-year LRFS was 100% in the HypoARC group *vs* 56% in patients treated with CRT. This shows that radiotherapy acceleration overcomes the ominus effect of a high cancer cell proliferation rate, which is in accordance with the concept that accelerated radiotherapy overcomes the effect of ‘accelerated repopulation’.

Similarly, HypoARC was more effective than CRT in tumours with p53 mutations. The 2-year LRFS was 100% in the HypoARC group *vs* 54% in the CRT group. Loss of wild-type p53 protein results in failure of radiation-induced DNA damage to trigger apoptosis events downstream p53 (Brugarolas *et al*, 1995). Large radiotherapy fractions that induce a more severe DNA damage may be more effective in wt-p53-deficient cells, which could explain the higher efficacy of HypoARC. HypoARC was also more effective in c-erbB-2 overexpressing tumours. Although a definite explanation for this finding cannot be provided, acceleration of radiotherapy may have overcome an eventual growth advantage conferred by c-erbB-2 upregulation (Mandler *et al*, 2000). Still, recent studies suggest that c-erbB-2-is involved in the repair of the radiation-induced DNA damage as MoAbs against c-erbB-2 enhance radiosensitivity of human breast and head–neck cancer cells (Pietras *et al*, 1999; Uno *et al*, 2001). Hypofractionation could therefore contribute to overcome an eventual c-erbB-2-dependent intrinsic radioresistance.

An interesting finding was that highly angiogenic tumours were resistant to radiotherapy, whether conventionally fractionated, accelerated or hypofractionated. This shows that altered fractionation or reduced overall treatment time are impossible to counteract the radioresistance pathways related to tumour angiogenicity. Antiangiogenic policies are awaited to improve the local control in patients with highly angiogenic breast cancer. Indeed, several experimental data suggest an important enhancement of the efficacy of radiotherapy when combined with various antiangiogenic agents (Gorski *et al*, 1999; Mauceri *et al*, 1998).

In a previous study, we showed that HypoARC is a regimen with reduced early toxicity and, at least within a median of 28 months of follow-up, late radiation sequelae were in the range of that expected from CRT. In the present study, we showed that HypoARC regimen is not at all equivalent to CRT, in terms of antitumour activity. It is evident that HypoARC, by using large radiotherapy fractions while accelerating radiotherapy, targets subgroups of breast tumours with a high risk to relapse following CRT. Tumours with a high proliferation index and eventually a high fraction of clonogens able to enter the phase of accelerated repopulation during CRT, as well as tumours with increased intrinsic radioresistance (e.g. mutations of p53 or c-erbB-2 gene amplification) have about 50% chance to recure after CRT; this seems to be averted using HypoARC. The ominous impact of upregulated angiogenic pathways, however, is unlikely to be prevented by altering the radiotherapy schedules. Antiangiogenic policies are demanded to improve local control. As HypoARC seems to overcome both intrinsic radioresistance and accelerated repopulation, taking also into account the promising low early and late side effects of the regimen, its role in clinical radiotherapy should be thoroughly examined. Prostate cancer, recently shown to have an *α*/*β* ratio lower than 2 Gy (Fowler *et al*, 2001), and also tumours with low intrinsic radiosensitivity (e.g. sarcomas, melanomas and pancreatic carcinoma) are expected to benefit from HypoARC regimens. The above findings should, however, be confirmed in larger studies, once mature data from ongoing HypoARC studies become available.
